# Promnesic, Anxiolytic and Antioxidant Effects of *Glaucosciadium cordifolium* (Boiss.) Burtt & Davis Essential Oil in a Zebrafish Model of Cognitive Impairment

**DOI:** 10.3390/plants12040784

**Published:** 2023-02-09

**Authors:** Razvan Stefan Boiangiu, Eyup Bagci, Gabriela Dumitru, Lucian Hritcu, Elena Todirascu-Ciornea

**Affiliations:** 1 Department of Biology, Faculty of Biology, Alexandru Ioan Cuza University of Iasi, 700506 Iasi, Romania; 2 Department of Biology, Faculty of Science, Firat University, 23119 Elazig, Turkey

**Keywords:** *Glaucosciadium cordifolium*, zebrafish, memory, anxiety, oxidative stress, scopolamine, Alzheimer’s disease

## Abstract

The purpose of this study was to investigate the effect of *Glaucosciadium cordifolium* essential oil (GCEO, 25 and 150 µL/L) on anxiety and learning and memory impairment induced by scopolamine (SCOP) in zebrafish. The chemical composition was analyzed by GC-MS, and the results showed that the highest content was limonene followed by α- and β-pinene, p-cymene and α-phellandrene. The dementia model was induced by SCOP (100 µM), whereas GCEO and galantamine (GAL, 1 mg/L) were delivered to the SCOP-induced model. It was found that GCEO significantly improved memory impairment and anxiety-like response induced by SCOP through the Y-maze, novel object recognition (NOR) test, and novel tank diving tests (NTT). Biochemical analyses showed that GCEO reduced SCOP-induced oxidative damage. Additionally, the cholinergic system activity was improved in the SCOP-induced model by decreasing the acetylcholinesterase (AChE) activity following the exposure to GCEO. It was clear that as a mixture, GCEO displays positive action in improving memory impairment through restoring cholinergic dysfunction and brain antioxidant status.

## 1. Introduction

Alzheimer’s disease (AD) is a progressive, unremitting brain disease and a major cause of dementia [1]. Due to damage of the neurons in the parts of the brain responsible for memory, language and thinking, the first AD symptoms tend to be memory loss and difficulties with thinking, language and problem-solving skills [1,2]. Besides these core symptoms of AD, neuropsychiatric symptoms, such as anxiety, apathy and depression, are frequently noted during the clinical course of the illness [3,4]. AD is characterized by extracellular accumulation of beta-amyloid plaques outside of the neurons, intraneuronal deposition of neurofibrillary tangles of protein tau and loss of cholinergic neurons in the forebrain along with a pronounce fall of acetylcholine (ACh) levels [5,6,7]. Cholinergic synapses are ubiquitous in the human central nervous system (CNS), and cholinergic transmission is considered to be critically important for memory, learning, attention and other higher brain functions [8]. Cholinergic transmission is achieved via nicotinic acetylcholine receptors (nAChRs) and muscarinic acetylcholine receptors (mAChRs), two families of ACh-binding receptors involved in cognition and affected in AD [9]. Scopolamine (SCOP) is an antagonist of mAChRs, and in neuroscience-related research, it is often used to induce cognitive disorders in experimental animals, as it readily crosses the blood–brain barrier. In the context of AD, SCOP causes cholinergic dysfunction and elevates amyloid-β deposition, both of which are hallmarks of the disease. Thus, SCOP is largely used to mimic a type of dementia noted in AD in experimental animals [10,11,12].

Zebrafish (*Danio rerio*) is a small, hardy freshwater fish that originated from India and has emerged as an excellent complementary model for neurodegenerative research due to the simplicity of the organism and robust and clearly visible behavior forms. Zebrafish is also a great translational model between in vitro and in vivo mammalian models for drug screening and for studying vertebrate development [13,14]. A high degree of similarity in terms of neuroanatomic and neurochemical pathways, along with psychological, emotional, and social behavioral patterns between zebrafish and humans, was evidenced [14,15]. Moreover, it has been shown that the zebrafish genome is evolutionarily conserved when it is compared to the human genome (almost 70% similarity) and that several genes orthologs of zebrafish are similar to those mutated in familial AD [13,14,15]. Hence, zebrafish models have been successfully used to simulate AD pathology [15,16].

The *Glaucosciadium* genus B.L. Burtt & P.H. Davis is represented by one taxon in Turkey and another two in the world [17,18]. In 1949, B.L. Burtt and P.H. Davis published *Glaucosciadium cordifolium* (syn. *Siler cordifolium* Boiss.) in *Kew Bulletin*. *G. cordifolium* is a monotypic plant that belongs to the *Umbelliferae* family, has a characteristic smell, grows on stony riverbanks and chalky slopes and is distributed in the Mediterranean region [17,19,20]. This species is known in Turkey as “sakarotu” or “çakşırotu”, and its roots and leaves has been used in traditional medicine for stomach ailments and as an aphrodisiac and appetizer [21,22]. Recently, *Glaucosciadium cordifolium* essential oil (GCEO) was proven to possess antimicrobial properties against *Listeria monocytogenes* [18] and antioxidant activities in different in vitro assays [17,18,19].

In the current study, we aimed to characterize *Glaucosciadium cordifolium* (Boiss.) essential oil from the aerial parts and to evaluate its anxiolytic, promnesic, anti-AChE and antioxidant potential in a SCOP-induced zebrafish model of cognitive impairment.

## 2. Results

### 2.1. Characterization of the Glaucosciadium Cordifolium Essential Oil

**Figure 1:**
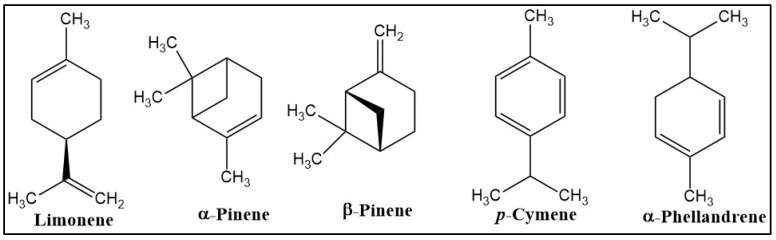
Chemical structure of the main identified compounds.

Following the GC-MS analysis, a total number of 22 compounds were detected in the GCEO, which represent approximately 98% of the total composition. Of these compounds, limonene (50.9%), α-(13.3%), β-pinene (10.5%), p-cymene (6.1%) and α-phellandrene (5.5%) were found in the greatest quantity (Figure 1).

All constituents identified in GCEO are listed in Table 1.

### 2.2. Effects on Memory and Anxiety-Like Behavior in Zebrafish

#### 2.2.1. The Zebrafish Performances in the NTT

The natural neophobic response of the zebrafish was evaluated with NTT. This behavior is defined as reduced exploration, increased freezing and/or unorganized irregular locomotion. In contrast, an increase in exploration, few freezing episodes and erratic movements are associated with a reduction in anxiety [23].

**Figure 2:**
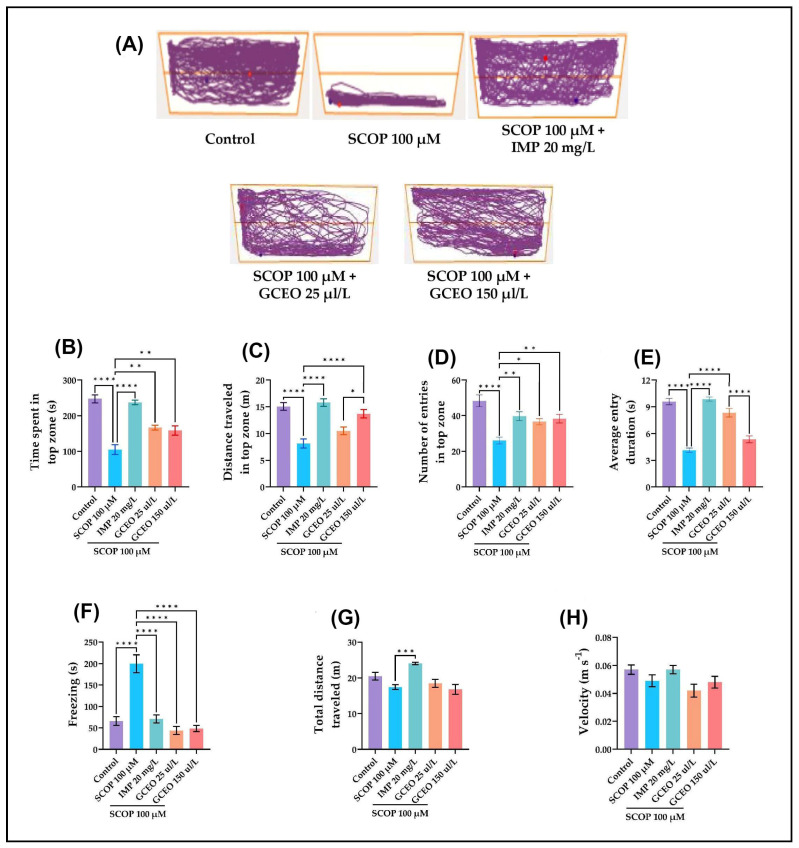
NTT results for *Glaucosciadium cordifolium* essential oil (GCEO, 25 and 150 µL/L). (**A**) The representative locomotion tracking patterns of the zebrafish in the NTT task. (**B**) Time spent in top zone (s). (**C**) Distance traveled in top zone (m). (**D**) Number of entries in top zone. (**E**) Average entry duration (s). (**F**) Freezing (s). (**G**) Total distance traveled (m). (**H**) Velocity (m^−1^s). Data are expressed as mean ± S.E.M. (*n* = 10). One-way ANOVA revealed overall significant differences between groups in (**B**) F(4,45) = 31.33, *p* < 0.0001, (**C**) F(4,45) = 18.38, *p* < 0.0001, (**D**) F(4,45) = 10.64, *p* < 0.0001, (**E**) F(4,45) = 52,94, *p* < 0.0001, (**F**) F(4,45) = 28.01, *p* < 0.0001 and (**G**) F(4,45) = 8.924, *p* < 0.0001. For Tukey’s post hoc analyses, **** *p* < 0.0001, *** *p* < 0.001, ** *p* < 0.01 and * *p* < 0.05.

After one week pre-treatment with 25 and 150 µL/L of GCEO, the anxiety-like behavior of the zebrafish was evaluated within NTT. The experimental model of cognitive impairment was induced by immersing the animals in SCOP 100 µM for 30 min before testing. The effects of SCOP and GCEO administration on anxiety-like behavior are depicted in Figure 2. Depending on the treatment, different swimming patterns of the zebrafish were observed. The representative tracking plots illustrated in Figure 2A indicate that the zebrafish treated with SCOP 100 µM exhibit intense activity at the bottom of the tank compared to the control group, thus suggesting an anxiogenic effect of SCOP. However, both groups treated with GCEO and the one treated with imipramine (IMP) show higher activity in the upper part of the tank. IMP, a tricyclic antidepressant, was used as a positive control in NTT, as it is also effective in reducing general anxiety [24]. The impact of GCEO treatment on anxiety-like behavior in zebrafish exposed to SCOP was evaluated in NTT by measuring the following parameters: the time spent in the top zone, the distance traveled in the top zone, the number of entries in the top zone, the average entry duration, and the freezing time. Tukey’s post hoc analyses revealed that SCOP treatment strongly reduced (*p* < 0.0001) the activity of the zebrafish in the top zone compared to the control group. Thus, the SCOP-treated zebrafish showed a reduction in time spent (Figure 2B), distance traveled (Figure 2C) and the number of entries (Figure 2D) in the upper half of the tank. In addition, SCOP significantly reduced the average entry duration (Figure 2E) and increased the freezing time of the zebrafish (Figure 2F). These data indicate that SCOP induces an anxiogenic effect in zebrafish within NTT. However, the SCOP-induced anxiogenic effect was significantly ameliorated by the coadministration of GCEO, especially when the higher dose was used. Hence, the GCEO-treated zebrafish spent more time (Figure 2B), traveled longer distances (Figure 1C) and accessed several times (Figure 2D) the top zone compared to the negative control. In addition, the treatment increased the average entry duration (Figure 2E) and lowered the freezing time (Figure 2F) of the zebrafish. These data suggest that GCEO administration exhibits anxiolytic properties in SCOP-treated zebrafish.

The impact of SCOP or GCEO treatment on locomotor activity was also investigated by measuring the total distance traveled (Figure 2G) and the velocity (Figure 2H) of the zebrafish. Therefore, SCOP alone or in combination with GCEO did not affect in any way the locomotion of the zebrafish, as there are no significant differences in total distance traveled or velocity between the SCOP group and those treated with GCEO.

#### 2.2.2. The Zebrafish Performances in the Y-Maze Task

**Figure 3:**
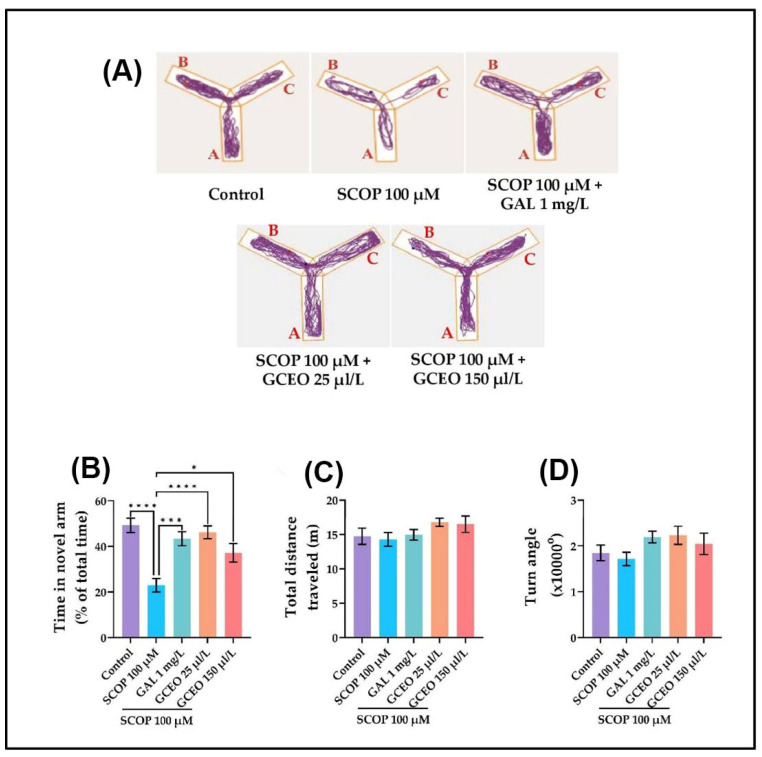
Y-maze task results for *Glaucosciadium cordifolium* essential oil (GCEO, 25 and 150 µL/L). (**A**) The representative locomotion tracking patterns of the zebrafish in the Y-maze task. (**B**) The time spent in the novel arm of the Y-maze. (**C**) Total distance traveled (m). (**D**) Turn angle (°). The start arm was marked with A, the other arm was marked with B, and the novel arm was marked with C. Data are expressed as mean ± S.E.M. (*n* = 10). One-way ANOVA revealed overall significant differences between groups in (**B**) F(4,45) = 10.32, *p* < 0.0001. For Tukey’s post hoc analyses, **** *p* < 0.0001, *** *p* < 0.001 and * *p* < 0.05.

The response to novelty and the spatial memory of the zebrafish were assessed within a Y-maze task. GCEO was chronically administered in two doses (25 and 150 µL) to SCOP-treated zebrafish, and their position in the Y-maze tank was evaluated. As illustrated in Figure 3A, different swimming patterns of the zebrafish were identified in the Y-maze tank according to their experimental group. The tracking plots of the SCOP-treated zebrafish revealed a reduced exploration of the novel arm compared to the control group. However, it appears that administration of GAL 1 mg/L or GCEO 25 and 150 µL/L to SCOP-treated zebrafish improved the exploratory behavior of the animals in the novel arm of the maze (Figure 3A). The spatial memory was assessed in the Y-maze test by measuring the time spent by the animals in the novel arm of the maze, and it was expressed as percentages of total time. As depicted in Figure 3B, the SCOP-exposed zebrafish spent almost 30% less time in the novel arm of the maze compared to the control group, thus suggesting a spatial memory impairment. The acute administration of GAL (1 mg/L) to the animals treated with SCOP significantly increased (*p* < 0.001) the time spent in the novel arm. GAL is a competitive, selective, and reversible inhibitor of AChE and is used in AD therapy to improve cognitive function. For this reason, GAL was use7d as a positive control in tests designed to evaluate cognition, such as Y-maze and NOR tasks. According to Figure 3B, both concentrations of GCEO, especially the lower one, significantly (*p* < 0.0001 for 25 µL/L and *p* < 0.05 for 150 µL/L respectively) increased the time spent by the animals in the novel arm of the maze compared to the group treated with SCOP alone. This behavior suggests that GCEO administration was able to ameliorate the SCOP-induced memory deficits in the zebrafish.

The locomotor activity was also investigated in the Y-maze assay by measuring the total distance traveled (Figure 3C) and the mean speed (Figure 3D) of the fish. Similar to the NTT test results, SCOP and GCEO treatment did not induce any alterations in the movement of the fish, as they traveled a distance and used a speed comparable with the control group.

#### 2.2.3. The Zebrafish Performances in NOR Task

**Figure 4:**
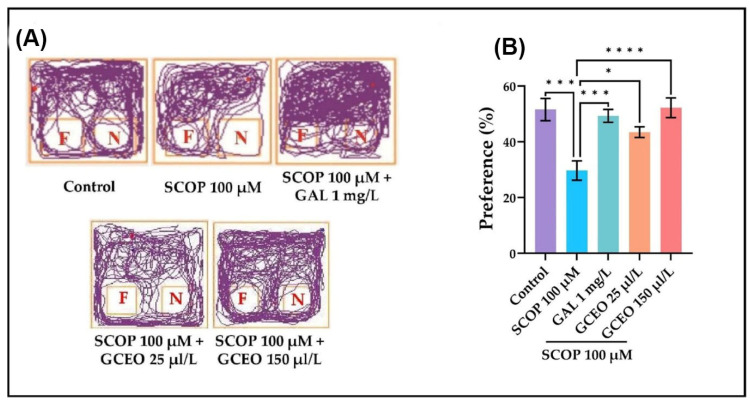
NOR task results for *Glaucosciadium cordifolium* essential oil (GCEO, 25 and 150 µL/L). (**A**) The representative locomotion tracking patterns of the zebrafish in the NOR task. (**B**) Preference (%). The familiar object (yellow cube) was marked with F, while the novel object (blue cube) was marked with N. Data are expressed as mean ± S.E.M. (n = 10). One-way ANOVA revealed overall significant differences between groups in (**B**) F(4,45) = 8.965, *p* < 0.0001. For Tukey’s post hoc analyses, **** *p* < 0.0001, *** *p* < 0.001, and * *p* < 0.05.

The recognition memory of the zebrafish was assessed by using the NOR task. The zebrafish can discriminate between a familiar and a novel object [25] and can recognize three-dimensional geometric shapes [26] from the environment. Based on this, we expressed the recognition memory as preference percentages. After performing the NOR test, we analyzed the representative locomotion tracking plots, which showed the path traveled by the fish according to their experimental group (Figure 4A). All fish initially exhibited thigmotaxis behavior, as they preferred to stay close to the edge, avoiding the central and open areas. The thigmotaxis was gradually reduced, and the fish accessed the central area of the tank in the testing session. In the plots corresponding to SCOP-treated zebrafish, we noticed that the animals exhibited a low exploration activity toward the novel object (N) compared to the familiar (F) one. The track plots also showed that fish treated with both SCOP and GCEO spent more time exploring N (Figure 4A). These behavioral observations were well associated with the calculated preference percentages. Hence, SCOP administration to zebrafish resulted in a lower exploration time of N and reduced by almost 21% the preference compared to the control group (Figure 4B). The lower preference percentage of this group indicates that SCOP caused recognition memory deficits in zebrafish. The acute administration of GAL was able to mitigate the SCOP-induced insults by significantly (*p* < 0.001) increasing the preference percentage. Similarly, and in a dose-dependent manner, GCEO ameliorated the SCOP-induced memory deficits by significantly (*p* < 0.05 for 25 µL/L and *p* < 0.0001 for 150 µL/L) increasing the exploration time of N and thus the preference percentage. These data show that GCEO exhibits a promnesic effect in a zebrafish model of cognitive impairment.

### 2.3. Effects on AChE Activity

**Figure 5:**
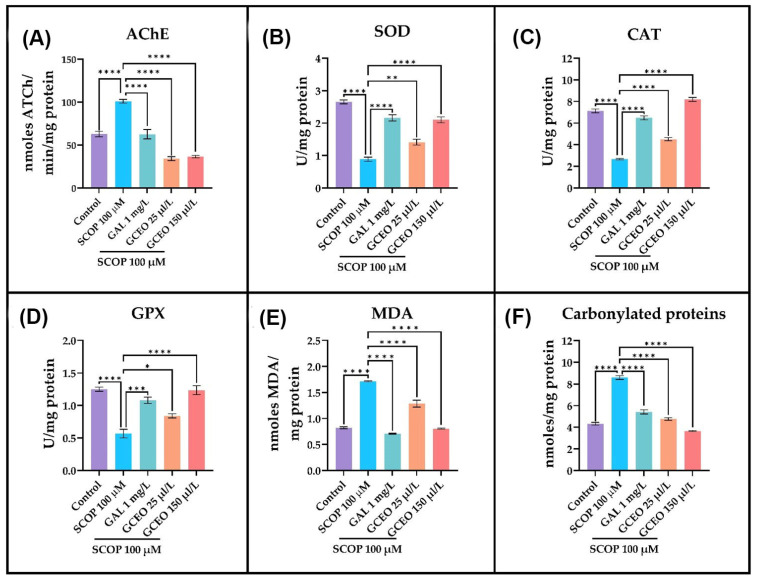
The effects of *Glaucosciadium cordifolium* essential oil (GCEO, 25 and 150 µL/L) on the specific activities of (**A**) AChE, (**B**) SOD, (**C**) CAT and (**D**) GPX and on the content of (**E**) MDA and (**F**) carbonylated proteins. The values are expressed as means ± S.E.M. (*n* = 3). One-way ANOVA revealed overall significant differences between groups in (**A**) F(4,10) = 71.34, *p* < 0.0001, (**B**) F(4,10) = 72.67, *p* < 0.0001, (**C**) F(4,10) = 198.1, *p* < 0.0001, (**D**) F(4,10) = 30.99, *p* < 0.0001, (**E**) F(4,10) = 177.8, *p* < 0.0001 and (**F**) F(4,10) = 185.4, *p* < 0.0001. For Tukey’s post hoc analyses, **** *p* < 0.0001, *** *p* < 0.001, ** *p* < 0.01 and * *p* < 0.05.

AChE belongs to the class of hydrolases, acting on ester bonds of esters of carboxylic acids (EC 3.1.1.7) [27]. AChE enzyme catalyzes the hydrolysis of the neurotransmitter ACh and breaks it down into choline and acetate ions. Overactivity of AChE leads to a deficiency of ACh in the synaptic cleft and thus the termination of synaptic transmission in the brain and degeneration of cholinergic system [27,28]. Thus, the use of AChE inhibitors represents a potential therapeutic strategy to limit the ACh degradation. In the current study, we evaluated the anti-AChE potential of GCEO in the SCOP-induced model of cognitive impairment. The specific activity of AChE was measured from the brain samples of the zebrafish and are expressed as nmoles of acetylthiocholine (ATCh) hydrolyzed/min/mg protein. According to Figure 5A, the administration of SCOP 100 µM significantly (*p* < 0.0001) increased the specific activity of AChE in the brain of the fish compared to the control group. As a well-known inhibitor of AChE, GAL diminished the enzymatic activity in SCOP-exposed zebrafish to a level close to the control group [29]. The effect of SCOP was also counteracted by GCEO, as both doses of 25 and 150 µL/L strongly reduced (*p* < 0.0001) the specific activity of AChE in the brain of the zebrafish. These findings indicate that GCEO possesses anti-AChE activity in an animal model of dementia.

### 2.4. Effects on Oxidative Stress

A contributing factor in the pathogenesis and progression of AD consists of oxidative stress. This phenomenon is defined as a pathophysiologic imbalance between oxidants, such as reactive oxygen species (ROS), and antioxidant defenses in favor of the former [30,31]. The impact of GCEO on oxidative status was evaluated in the SCOP-induced zebrafish model of cognitive impairment by measuring the specific activities of antioxidant defense enzymes, such as superoxide dismutase (SOD), catalase (CAT) and glutathione peroxidase (GPX), and the levels of malondialdehyde (MDA), the main product of lipid peroxidation, and carbonylated proteins, the product of protein oxidation. The acute exposure to SCOP 100 µM caused a significant decrease in the specific activities of SOD (*p* < 0.0001, Figure 5B), CAT (*p* < 0.0001, Figure 5C) and GPX (*p* < 0.0001, Figure 5D) enzymes in the brain of the zebrafish compared to the control group. In addition to reducing the activity of antioxidant defense enzymes, SCOP strongly increased the MDA (*p* < 0.0001, Figure 5E) and carbonylated protein (*p* < 0.0001, Figure 5F) levels, thus indicating elevated lipid and protein oxidation. These results indicate that SCOP induces oxidative stress in the brain of the zebrafish. Alternatively, the chronic administration of GCEO in SCOP-treated zebrafish significantly stimulated, in a dose-dependent manner, the specific activities of SOD (Figure 5B), CAT (Figure 5C) and GPX (Figure 5D) in the brain of the animals compared to the group treated with SCOP alone. Additionally, both concentrations of GCEO, especially the one of 150 µL/L, significantly lowered the MDA (Figure 5E) and carbonylated proteins (Figure 5F) levels in the brain of SCOP-exposed zebrafish. These data suggest that GCEO possesses antioxidant effects in a SCOP-induced animal model of cognitive impairment by stimulating the antioxidant defense enzymes and by decreasing lipid peroxidation and protein oxidation in the brain.

### 2.5. Correlations between Behavioral and Biochemical Parameters

The relationship between behavioral results and biochemical parameters was explored by using the Pearson correlation coefficient (*r*) [32]. We correlated the scores obtained from memory tasks, such as time spent in the novel arm (Y-maze test) and preference percentages (NOR test), and the specific activities of AChE and of the antioxidant defense enzymes, such as SOD, CAT and GPX, with MDA, the major product of lipid peroxidation. 

**Figure 6:**
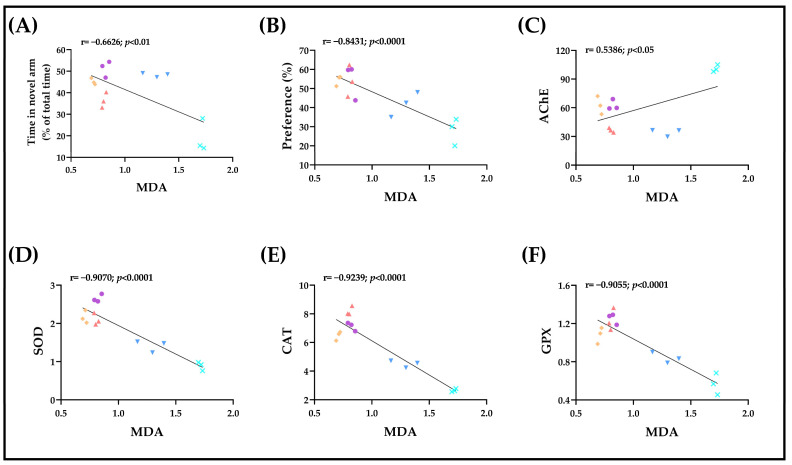
Pearson correlations between the behavioral and biochemical results. Data are expressed as time in novel arm (% of total time), preference (%), MDA (nmoles MDA/mg protein), AChE (nmoles acetylthiocholine (ACTh)/min/mg protein), SOD (U SOD/mg protein), CAT (U CAT/mg protein) and GPX (U GPX/mg protein). (**A**) Time in novel arm vs. MDA (*n* = 3, r = −0.662, *p* < 0.01), (**B**) preference vs. MDA (*n* = 3, r = −0.843, *p* < 0.0001), (**C**) AChE vs. MDA (*n* = 3, r = 0.538, *p* < 0.05), (**D**) SOD vs. MDA (*n* = 3, r = −0.907, *p* < 0.0001), (**E**) CAT vs. MDA (*n* = 3, r = −0.923, *p* < 0.0001) and (**F**) GPX vs. MDA (*n* = 3, r = −0.905, *p* < 0.0001) in control (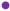), scopolamine (SCOP, 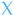), galantamine (GAL, 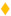), *Glaucosciadium cordifolium* essential oil (GCEO) 25 µL/L (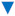) and 150 µL/L (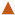) groups.

Firstly, we identified two correlations between the behavioral scores obtained from memory tasks and MDA. Our data indicate that the time spent by the zebrafish in the novel arm of the Y-maze is negatively correlated with the level of MDA in the brains of the animals (*n =* 3, *r =* −0.6626, *p* < 0.01, Figure 6A). Another significant negative correlation was also observed between the preference percentages calculated in the NOR task and MDA (*n =* 3, *r =* −0.8431, *p* < 0.0001, Figure 6B). These relationships indicate that an increase in MDA in the brain of the animals, and implicitly of oxidative stress, is well correlated with impairment of memory in behavioral tasks.

Secondly, Pearson analysis revealed a positive and significant correlation between the specific activity of AChE and the level of MDA (*n =* 3, *r =* 0.5386, *p* < 0.05, Figure 6C). This relationship suggests that AChE activity intensifies when the content of MDA is high in the brains of the animals.

Finally, three strong and negative correlations were identified between the specific activities of antioxidant defense enzymes and the content of lipid peroxidation product, MDA. Thus, an increase in the specific activities of SOD (*n =* 3, *r =* −0.9070, *p* < 0.0001, Figure 6D), CAT (*n =* 3, *r =* −0.9239, *p* < 0.0001, Figure 6E) and GPX (*n =* 3, *r =* −0.9055, *p* < 0.0001, Figure 6F) might lead to diminished oxidative stress and a decrease in MDA level in the brain.

## 3. Discussion

In this study, the composition of GCEO was elucidated by GC-FID/MS analysis. Our data showed that the major constituents of GCEO were limonene, α- and β-pinene, p-cymene and α-phellandrene. Our essential oil could be considered a good source of bioactive compounds (limonene, α- and β-pinene, p-cymene and α-phellandrene) responsible for the observed effects in the SCOP-treated zebrafish. In a previous study, GC-MS analysis revealed that GCEO contains monoterpene hydrocarbons (85.6%), and oxygenated monoterpenes (8.9%) constituted 95% of oil with limonene, α- and β-pinene as major components [20]. Additionally, 1.8% of the oil was represented by phthalides, such as (Z)-ligustilide and (E)- and (Z)-butylidene, which is also found in plants from the *Umbelliferae* family [20]. In another study, Karadağ et al. [18] analyzed, by GC-FID/MS, the GCEO obtained from the roots, fruits and aerial parts of the plant. They found a total of 62 volatile compounds, of which α-pinene was identified as the major component in all parts of the plant. The other main constituents were β-pinene, (Z)-β-ocimene and sabinene in the volatile oil of the aerial part, sabinene, β-pinene and α-phellandrene in the essential oil of the fruits and hexadecane, tetradecane and octadecane in the essential oil obtained from the root [18]. In a recent study, the GCEO composition from the fresh and dried stem, leaf and fruits was determined by GC-FID/MS [17]. It has been shown that the content of essential oil and its composition vary between the plant organs and whether they are fresh or dry. Thus, dried plant parts contained a higher content of essential oil. Carvacrol and α-pinene were the main components of the essential oil and were in higher amounts in fresh plant parts, while l-phellandrene and dl-limonene were in higher amounts in dry plant parts [17]. Based on the literature data, our essential oil has a chemical composition in concordance with previously described results. The impact of GCEO on memory and anxiety was evaluated in a zebrafish model of cognitive impairment induced by SCOP. Two doses of GCEO were chronically administered to SCOP-treated zebrafish and caused behavioral effects within in vivo tasks. Thus, our results showed that GCEO ameliorated SCOP-induced anxiety-like behavior in NTT and memory deficits in the Y-maze and NOR tasks. These results are in line with those from the literature and some of our studies, which showed that SCOP exposure causes different behavioral alterations, mainly depending on the dose and time of exposure [33,34,35,36]. In our previous studies, 30 min administration of SCOP 100 µM led to anxiety and spatial and recognition memory impairment [11,34,37]. 

The effect of GCEO on AChE-specific activity was also measured in the brains of SCOP-exposed zebrafish. Our findings indicate that the used doses of GCEO suppress the activity of AChE, which was elevated by SCOP exposure. Recently, Eruygur et al. [19] evaluated methanolic and aqueous extracts from different parts of *Glaucosciadium cordifolium*, such as roots, stem, leaves, and flowers, for their anti-AChE and anti-butyrylcholinesterase (BuChE) potential. Their results showed that all tested extracts displayed cholinesterase inhibitory activity. Of these, the aqueous extract of roots and leaves showed the greatest anti-AChE activity while the methanolic extract of stems exhibited the highest anti-BuChE activity [19].

The antioxidant potential of GCEO was also investigated in the SCOP-induced zebrafish model by measuring the specific activities of antioxidant defense enzymes and the extent of lipid and protein oxidation. Our data show that GCEO possesses antioxidant properties and reduces SCOP-induced oxidative stress by intensifying the SOD, CAT and GPX activities and by lowering the MDA and carbonylated proteins levels. Recently, the antioxidant properties of GCEO obtained from fresh and dried fruit, stem and leaf were investigated using the 2,2-diphenyl-1-picrylhydrazyl (DPPH) method [17]. It has been shown that essential oil from dry parts of the plant displays higher radical scavenging activity compared to fresh organs. The maximum radical scavenging activity values (32.70%) were obtained in the dry stem, while the lowest value (16.63%) was obtained in fresh leaves [17]. In another paper, the antioxidant effects of methanolic and aqueous extracts from various organs of *Glaucosciadium cordifolium* were investigated by using different in vitro assays: DPPH radical scavenging activity, 2,2′-Azinobis-(3-Ethylbenzthiazolin-6-Sulfonic Acid) (ABTS) radical scavenging activity, iron-chelating activity and β-carotene/linoleic acid emulsion method [19]. The results showed that these plant extracts exhibit high radical scavenging activity in these assays, and these antioxidant properties were attributed to their high amounts of polyphenols and flavonoids [19].

## 4. Materials and Methods

### 4.1. Chemicals

All chemicals were of the highest purity and were purchased from Sigma-Aldrich (Darmstadt, Germany), unless stated otherwise.

### 4.2. Plant Material Collection and Essential Oil Production

Aerial parts of *Glaucosciadium cordifolium* were collected in June 2019 from Tunceli, Turkey, and were dry-air dried at room temperature. Specimens (no. 2019HFU) were kept at the Herbarium of the Department of Biology, Firat University, Elazig, Turkey, and plant authentication was performed by Prof. Dr. Eyup Bagci. The essential oil was extracted by hydro-distillation with a yield of 0.7% (*v*/*w*) and stored at 4 °C until use.

### 4.3. Gas Chromatography–Mass Spectrometry (GC-FID/MS) Analysis

The essential oil of *Glaucosciadium cordifolium* was analyzed on an Agilent 5973N GS-MS system (Agilent Technologies, Santa Clara, CA, USA) equipped with 6890 GC with flame ionization detector (FID) [38]. The injector temperature was 250 °C, and the oven temperature increased from 70 to 240 °C at a rate of 5 °C/min. The sample was diluted in n-hexane (1:100, *v*/*v*) and carried by helium in 1 mL/min debit to a HP-5 MS column (30 m × 0.25 mm i.d., film thickness = 0.25 µm). The ionization energy was 70 eV with a mass range of 35–425. The chemical compounds identification was achieved by comparing their retention index to those of n-alkanes (C8–C22) as external references, their retention time (RT), and their mass spectra with those reported in MS libraries (Wiley) [39].

### 4.4. Laboratory Animals and the Outline of the Study

A total number of 60 adults (~4 months of age), both sexes (male:female 50:50 ratio), of wild-type, short-fin strain of zebrafish (*Danio rerio*) were purchased from an authorized commercial supplier (Pet Product S.R.L, Bucharest, Romania). The animals were housed in pairs of 10 in tanks filled with 30 L of dechlorinated water with well-established parameters: 27 ± 1 °C temperature, 7–7.2 pH, 7.2 mg O_2_/L, 1500–1600 µS/cm conductivity. The fish were fed twice daily with Norwin Norvital flake (Norwin, Gadstrup, Denmark) and were kept under 12 h light/dark cycle. The water was exchanged once every 3 days. The study was conducted according to the guidelines of the 2010/63/EU Directive of the European Parliament, and it was approved by the Ethics Committee on Animal Research of the Faculty of Biology, Alexandru Ioan Cuza University of Iași, Romania (no. 02/30.06.2020).

**Figure 7:**
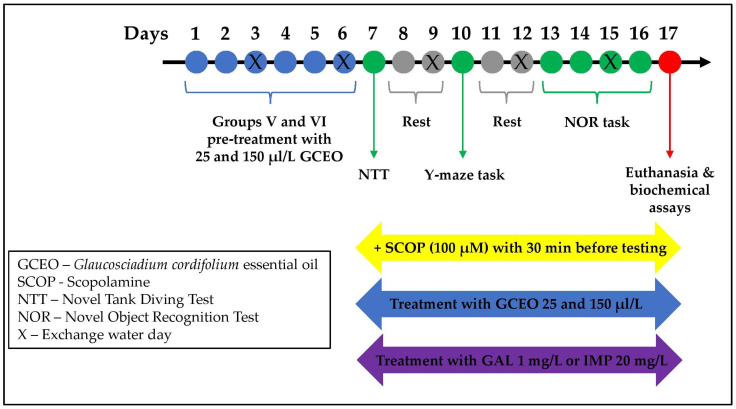
The experimental design of the study.

The animals were assigned to 6 experimental groups (10 fish/group) as follows: (I) Control; (II) SCOP (fish treated with 100 µM SCOP); (III) SCOP + GAL 1 mg/L (fish treated with 100 µM SCOP and 1 mg/L GAL); (IV) SCOP + IMP 20 mg/L (fish treated with 100 µM SCOP and 20 mg/L IMP); (V) SCOP + GCEO 25 µL/L (fish treated with 100 µM SCOP and 25 µL/L GCEO); (VI) SCOP + GCEO 150 µ/L (fish treated with 100 µM SCOP and 150 µL/L GCEO). Groups I, II, III and IV received 1% Tween 80 as vehicle. GCEO was prepared in 1% Tween 80 solution and delivered to zebrafish by immersion in home tank water in final concentrations of 25 and 150 µL/L. All doses were selected based on the previous studies of our group [40,41]. The treatment was administered once for 17 consecutive days (7 days pre-treatment and 10 days during behavioral assessment, Figure 7). The zebrafish model of AD was induced by adding the fish to 100 µM SCOP solution for 30 min before starting each behavioral test. After SCOP administration, groups III and IV were treated acutely, for 3 min before testing, with GAL 1 mg/L and IMP 20 mg/L, respectively, and were considered the positive controls of our study (Figure 7).

### 4.5. Behavioral Tasks

The swimming behavior of the zebrafish within in vivo assays was recorded using a Logitech C922 Pro HD Stream camera (Lausanne, Switzerland), and the recordings were analyzed with ANY-maze software v6.3 (Stoelting Co., Wood Dale, IL, USA).

#### 4.5.1. Novel Tank Diving Test (NTT)

NTT was used to specifically measure the anxiety-like behavior of the zebrafish as was previously described by Rosemberg et al. [42] and Cachat et al. [43]. The fish were placed individually in a trapezoidal-shaped glass tank (23.9 cm along the bottom × 28.9 cm at the top × 15.1 cm high with 15.9 cm along the diagonal side, 7.4 cm wide at the top and 6.1 cm wide at the bottom) filled with 1.5 L of home tank water. The behavior was recorded for 6 min with a camera placed at 40 cm in front of the tank, which was virtually divided into two zones: top and bottom. The anxiety-like behavior was expressed as time spent in top zone (s), distance traveled in top zone (m), number of entries in top zone, average entry duration (s) and freezing (s). Additionally, locomotor activity was measured using the total distance traveled (m) and the velocity (m/s).

#### 4.5.2. Y-Maze Test

The Y-maze test was used to assess the zebrafish’s response toward novelty. The Y-maze test protocol was previously described by Cognato et al. [44] and measures spatial recognition memory of the zebrafish. The test was performed in a glass Y-shaped aquarium of 3 L with three arms in sizes of 25 × 8 × 15 cm (L × l × h), which radiates from a central equilateral triangle. The arms of the maze were marked on the outside with different visual cues, such as white circles, triangles or squares on a black background, and were named as follows: the start arm (A), the other arm (B) and the novel arm (C). The protocol was divided in two sessions, namely, the training session and the testing session, separated by a 1 h retention interval. In the training session, the novel arm (C) was kept closed, the fish was placed individually in the start arm (A) and was allowed to explore the maze (both the start arm and the other arm (B)) for 5 min. In the testing session, the novel arm was opened and the fish were placed in the start arm and allowed to freely explore all three arms of the maze for 5 min. The spatial recognition memory was evaluated by measuring the time spent by the fish in the novel arm (% of total time) during the testing session. The locomotor activity was assessed by measuring the total distance traveled (m) and the absolute turn angle (°).

#### 4.5.3. Novel Object Recognition Test

The recognition memory of the zebrafish, i.e., the ability of these to recognize novel objects in the environment, was assessed in the novel object recognition (NOR) test, as previously described by Gaspary et al. [45] and Stefanello et al. [46]. The test was conducted in a glass aquarium of 30 × 30 × 30 cm and filled with a water column of 6 cm. External interference and reflections were avoided by covering the tank with a piece of black cloth. The test protocol was divided into three different sessions: the habituation session (T_0_), the training session (T_1_) and the testing session (T_2_). The animals were habituated individually to the empty tank for three consecutive days, twice per day (2 trials/day with 5 h period between trials) for 5 min. On the fourth day and within T_1_, the fish were placed in the tank and allowed to explore two identical objects (two yellow cubes) for 10 min. Post-training, the animals were subjected to a 1 h retention interval, in which SCOP, GAL and IMP were administered. In T_2_, one of the familiar objects (yellow cube, F) was replaced with a new one (blue cube, N), and the fish were allowed to explore both objects for 10 min. The recognition memory was expressed as preference % and was calculated using the following formula: [time spent exploring N/(time spent exploring F + time spent exploring N)] × 100. The cubes had sides of 2.5 cm in length, were made of non-toxic plastic and were colored in yellow or blue, as the zebrafish show no innate preference for these colors [47]. The exploring area was established before as increasing the size of the object area [48]. Therefore, exploratory behavior was considered when the fish were located at no more than 2.5 cm on either side of the object.

### 4.6. Biochemical Analysis

Once the behavioral tasks were finished, the zebrafish were euthanized by rapid cooling. It is known that this method avoids the biochemical and physiological alterations that could prevent post-mortem analysis [49]. The animals were immersed in ice-cold water (2–4 °C) until no opercular or tail movements were observed. Subsequently, the whole brain was carefully dissected according to the procedure described by Gupta and Mullins [50] and collected for biochemical analysis. Two brains were pooled and considered as an independent sample. The brains were homogenized (1:10 ratio, *w*/*v*) in ice-cold 0.1 M potassium phosphate buffer with 1.15% KCl (pH 7.4), for 1 min at 1000 rpm using the Mikro-Dismembrator U mill (Sartorius, New York, NY, USA) equipped with 3 mm diameter magnetic balls (Sartorius Stedim Biotech GmbH, Goettingen, Germany). Then, the crude homogenates were centrifuged for 15 min at 14,000 rpm and 4 °C, and the supernatants were further used to measure the total content of soluble proteins (bicinchoninic acid assay) [51], the activities of SOD (EC 1.15.1.1) [52], CAT (EC 1.11.1.6) [53], GPX (EC 1.11.1.9) [54], AChE (E.C. 3.1.1.7) [55] and the levels of MDA [56] and carbonylated proteins [57].

### 4.7. Statistical Analysis

The data were expressed as means ± standard error of mean (S.E.M.) and were statistically analyzed with GraphPad Prism v9.4 software (La Jolla, CA, USA) using one-way analysis of variance (one-way ANOVA) and Tukey’s post hoc multiple comparison tests. Statistically significant differences were considered when *p* < 0.05. Pearson correlation coefficient (r) was applied to correlate the behavioral and biochemical results.

## 5. Conclusions

The current study was conducted to investigate the promnesic, anxiolytic and antioxidant potential of GCEO in a zebrafish model of cognitive impairment induced by SCOP. Our data demonstrated that GCEO administration mitigated the memory deficits and the anxiety-like behavior measured in the specific in vivo tasks. In addition, the observed effects could be attributed to the main identified compounds such as limonene, α- and β-pinene, p-cymene and α-phellandrene. Moreover, the chronic treatment with GCEO reduced the SCOP-induced oxidative stress and displayed a pronounced anti-AChE effect. This study suggests, for the first time, that GCEO possesses promnesic, anxiolytic and antioxidant effects that could be used effectively in amelioration of dementia-related conditions.

## Figures and Tables

**Table 1:** The composition of *Glaucosciadium cordifolium* essential oil determined by GC-MS analysis.

No.	Constituents of *Glaucosciadium cordifolium* Essential Oil ^a^	RI ^b^	Area (%) ^c^
1.	α-Pinene	1032	13.3
2.	α-Thujene	1035	1.4
3.	Camphene	1076	0.2
4.	β-Pinene	1118	10.5
5.	Sabinene	1132	0.9
6.	β-Myrcene	1174	2.0
7.	α-Phellandrene	1176	5.5
8.	Limonene	1203	50.9
9.	β-Phellandrene	1218	1.7
10.	β-Ocimene	1246	2.0
11.	γ-terpinene	1255	1.7
12.	p-Cymene	1280	6.1
13.	Terpinolene	1290	0.5
14.	*trans*-1,2-Limonene epoxide	1468	0.2
15.	Terpinen-4-ol	1611	0.5
16.	β-Caryophyllene	1612	0.1
17.	Germacrene D	1726	0.1
18.	p-Mentha 1(7), 5-dien-2-ol	1823	0.1
19.	β-Damascenone	1835	0.1
20.	Caryophyllene oxide	2008	0.2
21.	Spathulenol	2144	0.2
22.	Carvacrol	2240	0.2
	**Total**		**98.4**

^a^ The constituents are listed in their order of elution from HP-5 MS column. ^b^ Experimental retention index relative to standard mixture of n-alkanes on HP-5 MS column. ^c^ Relative peak area percentage (average of three determinations).

## Data Availability

The data presented in this study are available on request from the corresponding author.
